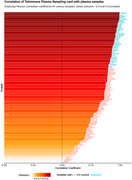# Multiplex Biomarker Detection in Dried Plasma Spots: finding the best biomarker for remote blood collection

**DOI:** 10.1002/alz70856_106925

**Published:** 2026-01-11

**Authors:** Jakub Vávra, Wiebke Traichel, Andrea Benedet, Hanna Huber, Kaj Blennow, Laia Montoliu‐Gaya, Nicholas Ashton, Henrik Zetterberg

**Affiliations:** ^1^ Institute of Neuroscienace and Physiology, University of Gothenburg, Mölndal, Västra Götaland, Sweden; ^2^ German Center of Neurodegenerative Diseases (DZNE), Bonn, North Rhine‐Westphalia, Germany; ^3^ Paris Brain Institute, ICM, Pitié‐Salpêtrière Hospital, Sorbonne Université, Paris, France; ^4^ Neurodegenerative Disorder Research Center, Division of Life Sciences and Medicine, Institute on Aging and Brain Disorders, University of Science and Technology of China and First Affiliated Hospital of USTC, 合肥, 安徽, China; ^5^ Clinical Neurochemistry Laboratory, Sahlgrenska University Hospital, Mölndal, Sweden; ^6^ King's College London, Institute of Psychiatry, Psychology & Neuroscience, Maurice Wohl Clinical Neuroscience Institute, London, United Kingdom; ^7^ Banner Health Foundation & Banner Alzheimer's Foundation, Phoenix, AZ, USA; ^8^ Clinical Neurochemistry Laboratory, Sahlgrenska University Hospital, Mölndal, Västra Götalands län, Sweden; ^9^ Department of Neurodegenerative Disease, UCL Queen Square Institute of Neurology, University College London, London, ‐, United Kingdom; ^10^ Wisconsin Alzheimer's Disease Research Center, University of Wisconsin‐Madison, School of Medicine and Public Health, Madison, WI, USA; ^11^ Hong Kong Center for Neurodegenerative Diseases, Hong Kong, China; ^12^ UK Dementia Research Institute at UCL, London, United Kingdom

## Abstract

**Background:**

Conventional blood sampling for the testing of Alzheimer's disease (AD) biomarkers depends on stringent, time‐sensitive, and temperature‐dependent protocols for processing, shipping, and storage. Dry plasma spots (DPS) present a simpler, more scalable alternative for the collection, storage, and transport of blood samples and may offer an alternative sampling when access to blood volume is limited. Notably, neurodegenerative biomarkers such as *p*‐tau, NfL, and GFAP from DPS have demonstrated a strong correlation with paired plasma on other platforms. In this pilot study, we aimed to expand on these findings by exploring a broader panel of central nervous system (CNS) biomarkers using DPS, assessing their potential for reliable and accurate detection.

**Method:**

We used the NULISA™ platform to test multiplex detection of a CNS biomarker panel (127 proteins) in DPS and matched plasma, examining plasma–DPS correlations. A discovery cohort (*n* = 14; mean age 71.1 ± 12.8 years; 8 females [57%]) was selected from the Clinical Neurochemistry Laboratory in Mölndal, Sweden. DPS (Telimmune™ Plasma Separation Card) spiked with venous blood, were analysed with their paired EDTA plasma collected by traditional venipuncture. Pearson correlation was used to compare protein quantification across sample types.

**Result:**

We demonstrated several biomarker associations between DPS and plasma with a correlation coefficient >0.99 and *p* <0.0001 (Figure 1), including APOe4 (*r* = 0.996), IL6 (*r* = 0.995), and FABP3 (*r* = 0.994). Notably, AD‐related biomarkers like *p*‐tau181 (r=0.89), *p*‐tau231 (r=0.86), GFAP (r=0.8), NPTX2 (r=0.92), NFL (r=0.95), SMOC1 (r=0.91), and total Tau (r=0.93) all showed strong correlations and *p* <0.0001. DOPA decarboxylase, relevant for LBD and atypical Parkinsonian disorders, also correlated strongly (r=0.98, *p* <0.0001). VGF, a biomarker of synaptic plasticity altered in AD and Major Depressive Disorder showed a strong correlation (*r* = 0.95, *p* <0.0001). Among 16 interleukins, 11 had r>0.8 (*p* <0.0003) and 4 had r>0.5 (*p* <0.05), with IL6 (r=0.995) and IL12 (r=0.994) correlating notably strong (*p* <0.0001). However, 25% of proteins have a weak correlation coefficient of r<0.5 with plasma.

**Conclusion:**

Our findings highlight the potential of DPS as a practical and scalable tool for multiplex biomarker detection. Further research is required to identify and validate optimal AD biomarkers in DPS‐based multiplex assays.